# Can One Define the Conductance of Amino Acids?

**DOI:** 10.3390/biom9100580

**Published:** 2019-10-07

**Authors:** Linda A. Zotti, Beatrice Bednarz, Juan Hurtado-Gallego, Damien Cabosart, Gabino Rubio-Bollinger, Nicolas Agrait, Herre S.J. van der Zant

**Affiliations:** 1 Departamento de Física Teórica de la Materia Condensada, Universidad Autónoma de Madrid, Ciudad Universitaria de Cantoblanco, E-28049 Madrid, Spain; 2 Condensed Matter Physics Center (IFIMAC), Universidad Autónoma de Madrid, Ciudad Universitaria de Cantoblanco, E-28049 Madrid, Spain; 3 Kavli Institute of Nanoscience, Delft University of Technology, Lorentzweg 1, 2628 CJ Delft, The Netherlands; 4 Departamento de Física de la Materia Condensada, Universidad Autónoma de Madrid, Ciudad Universitaria de Cantoblanco, E-28049 Madrid, Spain; 5 Instituto Madrileño de Estudios Avanzados en Nanociencia (IMDEA Nanociencia), Campus Universitario de Cantoblanco, E-28049 Madrid, Spain

**Keywords:** break junctions, DFT, NEGF, amino acids, electron transport, biomolecular electronics

## Abstract

We studied the electron-transport properties of ten different amino acids and one dimer (di-methionine) using the mechanically controlled break-junction (MCBJ) technique. For methionine and cysteine, additional measurements were performed with the scanning tunneling microscope break-junction (STM-BJ) technique. By means of a statistical clustering technique, we identified several conductance groups for each of the molecules considered. Ab initio calculations revealed that the observed broad conductance distribution stems from the possibility of various binding geometries which can be formed during stretching combined with a multitude of possible conformational changes. The results suggest that it would be helpful to explore different experimental techniques such as recognition tunneling and conditions to help identify the nature of amino-acid-based junctions even further, for example, with the goal to establish a firm platform for their unambiguous recognition by tunneling break-junction experiments.

## 1. Introduction

The electron-transport properties of proteins and peptides have recently attracted enormous interest in light of their potential as active elements in solid-state devices and possible use in biomedical applications [1,2]. In particular, striking results have been demonstrated, indicating efficient long-range charge transport [2]. However, the exact transport mechanism in these systems is still under debate and considerable effort is currently being made in order to clarify this issue [3,4,5,6].

To this aim, it would be helpful to understand the fundamental transport properties of amino acids, as they are the building blocks of peptides and proteins. For instance, the difference in the electronic structure among different amino acids has been claimed to affect the conductance properties of self-assembled monolayers of peptides [7]. Recent theoretical work has suggested that conductance measurements based on single-molecule techniques can help in gaining additional insight into the electron-transport mechanism of these systems [8,9]. Prior to this work, scanning tunneling microscopy break-junction (STM-BJ) measurements were performed on several amino acids [10,11], and measurements based on recognition tunneling [12] and nanopore sequencing [13] on selected species have shown remarkable capabilities in identifying amino acids. However, to date, a systematic study aimed at understanding the electron-transport properties of these molecules via combined experimental and theoretical observations for a broad series is still lacking. Indeed, from the theoretical perspective, extensive studies have been focused on the adsorption of amino acids on surfaces [14] or nanotubes [15] and their chemical reactivity [16], but little has been done to study the behavior of these molecules when trapped between two gold electrodes [17]. Furthermore, the STM-BJ measurements [10,11] gave conflicting results concerning the conductance values of alanine, which could be due to the use of different environments: aqueous solution in the former and ambient in the latter case.

**Figure 1:**
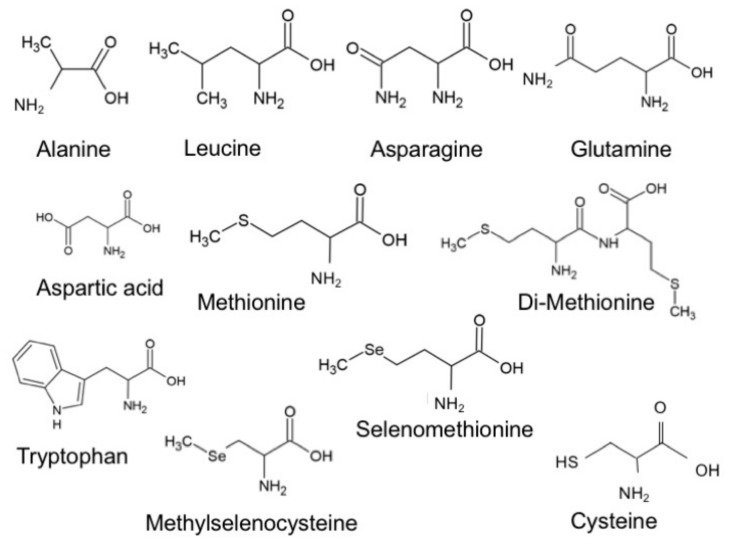
The 10 amino acids and one dimer (di-methionine) analyzed in this work.

In this work, we carried out a systematic study on molecular junctions based on ten amino acids (see Figure 1) and performed ab initio theoretical calculations to rationalize the experimental results. In nature, 20 different amino acids are present, which can be expressed (except for proline) with one general formula, R-CH(NH2)COOH, where R is the side chain of the amino acid. In the present study, we analyzed the following molecules: asparagine, leucine, glutamine, cysteine, methionine, alanine, tryptophan, aspartic acid, selenomethionine di-methionine, and methylselenocysteine. These amino acids were chosen to study the influence of different anchoring groups (e.g., methionine, cysteine, selenomethionine, methylselenocysteine, leucine, glutamine, aspartic acid), their length while having the same anchoring groups (e.g., asparagine and glutamine differ in length by one carbon atom), and the presence of an aromatic ring that can interact with the gold electrodes (tryptophan). Di-methionine was chosen to assess if in situ dimerization in the methionine measurement would play a role. We show that these systems display an interesting variety of conductance values; multiple metal–molecule binding possibilities as well as conformational distortions and environmental influences may all contribute to the variability present in the experiment.

## 2. Materials and Methods 

### 2.1. Experiments

**Figure 2:**
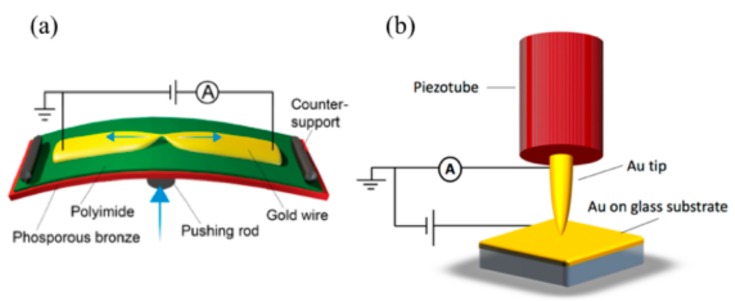
(**a**) Sketch of the mechanically controlled break junction MCBJ setup (from Reference [23]). The sample is bent and consequently broken by moving the pushing rod up; (**b**) schematic representation of the scanning tunneling microscopy break-junction (STM-BJ) setup. The motion of the tip is controlled by the piezotube [20].

For the conductance measurements, two different break-junction techniques [18] were employed: a scanning tunneling based one (STM-BJ; Madrid Figure 2b) and a mechanically controlled break-junction technique (MCBJ; Delft). The two techniques exhibit different electrode shapes—flat surface and sharp tip (STM-BJ) versus two sharp tips (MCBJ)—so that the geometrical influences on the conductance may be probed. In the STM-BJ [19] approach, we employed a home-built STM [20] with annealed 250 nm thick gold Au(111) film on glass (ArrandeeTM, Germany) as substrates and mechanically cut Au wires (0.25 mm in diameter, 99.99% purity) as tips. Nanometer-sized contacts were formed by indenting the substrate with the STM tip. The STM-BJ measurements were performed in ambient conditions and at room temperature, with a bias voltage of 0.1 V applied to the substrate.

**Figure 3:**
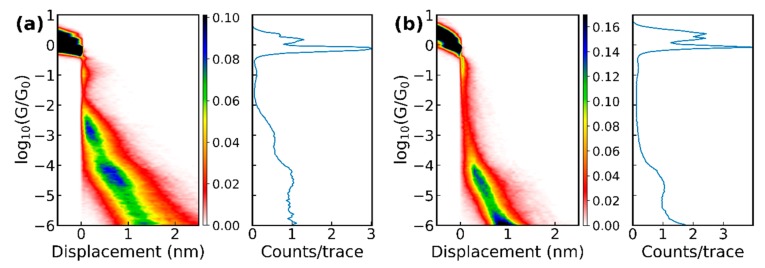

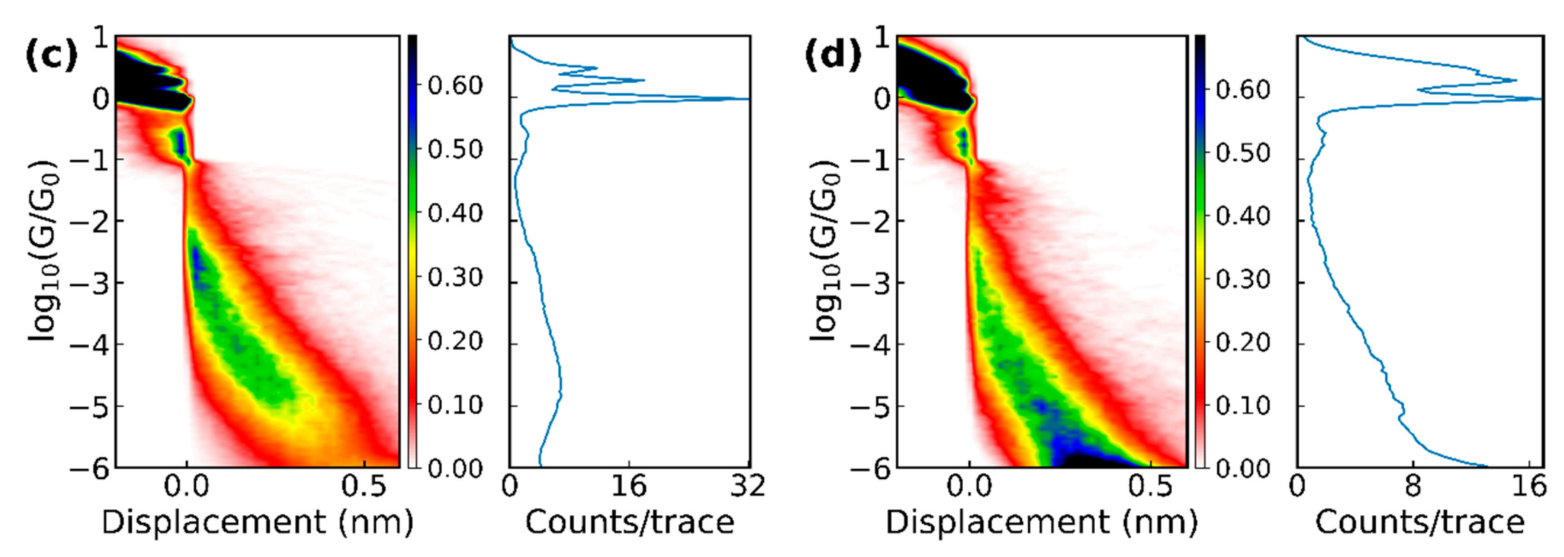
1D and 2D histograms of the raw data of (**a**) 6000 breaking traces of methionine and (**b**) 4000 breaking traces of cysteine measured with the MCBJ setup, as well as (**c**) 5745 traces of methionine and (**d**) 3584 traces of cysteine at pH = 7 measured with the STM-BJ.

In the MCBJ technique [21], a bendable phosphorous bronze substrate was used, coated with a thin insulating layer of polyimide. Four gold wires with a constriction of approximately 40 nm in width were patterned on top of the polyimide using electron-beam lithography and lift-off. To suspend the constriction for improved breaking of the junction, the samples were exposed to an O_2_–CF_4_ plasma. Directly before measuring, they were cleaned in an ozone treatment. After loading a sample, we recorded 2000 conduction versus displacement curves of pure gold by breaking and merging a junction successively at 0.1 V bias voltage (see Figure 3). Gold samples that we identified as clean in this way were used to measure the conduction through the amino acids. 

The amino acids were obtained from different suppliers (see the list in Supplementary Materials Section 6) as powders and dissolved in demineralized water with a concentration between 0.05 and 1.0 mM. For the STM-BJ experiments, the amino acids were deposited by drop-casting onto the gold substrate after the annealing and then dried with streaming nitrogen. For the MCBJ experiments, we added 5 ± 2.5 µl onto a clean junction and let the junction dry. After the water had evaporated, conductance versus displacement breaking traces were measured in both setups; the electrode displacement speed was 1–2 nm/s (MCBJ) or 6 nm/s (STM-BJ). Plotting these breaking traces for a molecule as 2D and 1D histograms gives an overview of the possible plateau positions, which are generally viewed as representing the more stable junction conformations or configurations. Figure 3 displays the conductance histograms for methionine and cysteine obtained with both break-junction techniques. The 1D histograms showed the number of counts for a certain conductance averaged over all traces. In these histograms, the 1G_0_ plateau showed that only a single bond between the two gold electrodes was left.

By performing an unsupervised clustering analysis [22], we improved the visibility of the plateaus of the different amino acids, facilitating a more detailed characterization. More precisely, the breaking traces were sorted into five groups, so called *classes*, according to their form and individual 1D histogram with the *K*-means algorithm (see appendix for details and a comparison of the results when the number of classes was changed). For every group with a plateau, the average conductance value, length, and slope were determined.

### 2.2. Theoretical Calculations

We performed theoretical calculations using a combination of density functional theory (DFT) and non-equilibrium Green’s function techniques (NEGF), as explained in detail in Reference [24] and based on the quantum chemistry package Turbomole [25]. The structures of all amino acids were first optimized in the gas phase and then placed between two gold clusters of 20 atoms for all cases, except for the geometries similar to “d” and “i” of Figure 6, in which the C terminal of the amino acids (that containing the –COO group) was connected to a 19 atom cluster. The so-built structures were then optimized by relaxing the molecule and the atoms in the three closest gold layers. The gold structures were subsequently extended to 63 atoms in order to provide the correct metal–gold charge transfer by increasing the accuracy of the energetic position of the gold Fermi level. This step is a well-corroborated technique [26,27] based on previous test calculations on molecular junctions which showed that employing even larger gold clusters (up to 120 atoms) did not change the energetic alignment of the molecular orbitals with respect to the Fermi level. 

For the calculations in this work, a def-SVP basis set [28] and the BP86 [29] exchange-correlation functional were used. Finally, the total transmission, τ, was computed in the spirit of the Landauer formalism. The low-temperature conductance was given by G = G_0_τ(E_F_) = G_0_Σ_i_ τ_i_(E_F_), where G_0_ is the conductance quantum 2e^2^/h, E_F_ is the Fermi energy, and {τ_i_} are the transmission coefficients.

## 3. Results and Discussion

### 3.1. Experiments

With the MCBJ technique, all 10 amino acids and the methionine dimer were measured; and the STM-BJ measurements were performed on methionine and cysteine, the latter one prepared in a solution with a pH of 7 and 10. For all molecules, next to breaking traces that showed pure tunneling (no molecule trapped inside the junction), “plateau”-like features in the conductance versus displacement curves were observed (some examples of individual traces can be found in the Supplementary Materials Figure S1). A common feature of the measurements is that the plateaus were generally short (few tenths of a nanometer) and that, in some cases, more than one plateau could be found in an individual trace, indicating the presence of different binding configurations while moving the electrodes further apart. The 2D histograms of the data (see Figure 3) with amino acids showed clear differences with respect to the histograms of pure gold. However, clear plateaus in the conductance histograms were difficult to distinguish because of their short length, the large variation in conductance they covered, and because they typically were not completely horizontal. 

**Figure 4:**
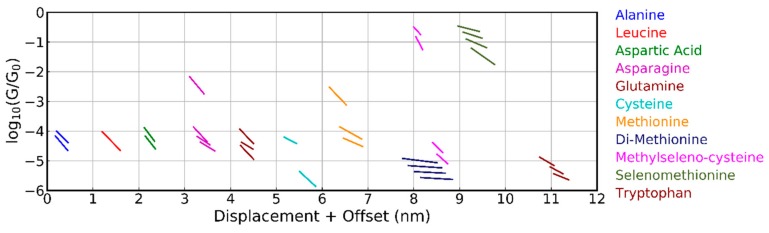
Overview of the average conductance plateaus for all amino acids measured with the MCBJ, obtained from the clustering analysis into five classes. Plateaus are only shown, if they are shorter than the molecule and less steep than the tunneling slope. The plateau positions for subsequent molecules are offset by 1 nm in the horizontal displacement axis.

A clustering analysis (Section 2 and Supplementary Materials) was employed to enhance the visibility of the different plateaus. We first discuss the results obtained with the MCBJ technique. When using five classes, the average plateau positions are displayed in Figure 4; clear differences among the behavior of the different molecules were seen. For leucine, only one class was found, while for the other molecules, up to four different classes could be distinguished. Most of the plateaus were between 10^‒4^ and 10^‒5^ G_0_ and were found at an average displacement of about 0.5 nm. For alanine and aspartic acid, two groups were found at similar conductance values, which were at least one order of magnitude lower than those obtained with the STM-BJ technique [10,11], albeit without clustering the data. The high conduction around 0.1 G_0_ was through selenomethionine and methylselenocysteine which share a seleno-ether group. The reason for this high conduction is not understood.

The amide and thio-ether group of asparagine and methionine had similar conductance plateaus with the highest one between 10^‒2^ and 10^‒3^ G_0_. Glutamine and cysteine did not show this plateau, although they have very similar anchoring groups. The influence of a single carbon atom can be analyzed by comparing asparagine and glutamine, as well as methylselenocysteine and selenomethionine; both pairs, however, did not show a clear shift in conductance. Only tryptophan, cysteine, and di-methionine showed conductance plateaus below 10^‒5^ G_0_; these appeared at larger electrode displacements. A more detailed analysis of the methionine and di-methionine data (additional clustering performed on the combined set of data; not shown in Figure 4) showed that di-methionine had all of the conductance plateaus of methionine, as well as some extra plateaus at low conductance and larger displacements (those are the one that show up in the cluster analysis of Figure 4). This observation indicates that dimerization in the experiment apparently does not play an important role. Finally, we note that all molecules had at least one class of approximately 30% of the breaking traces in the clustering analysis where no plateau could be identified; in those cases, a molecule was bound inside the junction.

**Figure 5:**
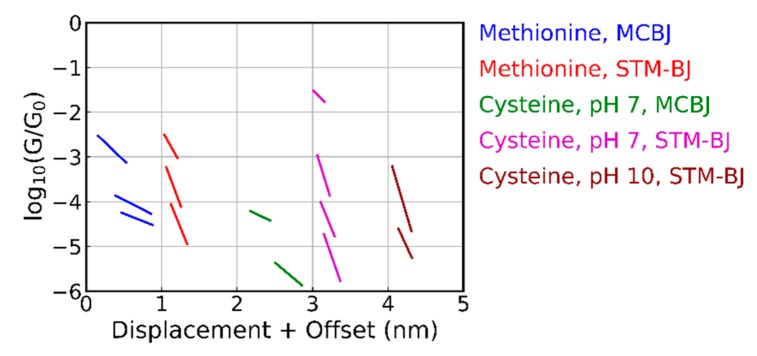
Comparison of the average conductance plateaus for methionine and cysteine obtained from the clustering analysis into 5 classes as measured by the two break-junction techniques. Plateaus (slanted line segments) are only shown if they are shorter than the amino acid and less steep than the tunneling slope. The plateau positions for subsequent molecules are offset by 1 nm in the horizontal displacement axis. The MCBJ data are the same as in Figure 4.

In Figure 5, the results for methionine and cysteine obtained with the two break-junction techniques and analyzed using the same clustering algorithm are compared. Plateaus appeared at approximately the same conductance values indicating that similar junction configurations were probed in the two setups; for the STM-BJ experiment on cysteine (pH 7), additional plateaus at higher conductance values were found. Possibly, the snap-back distance when breaking the final gold atoms may be smaller in the case of the STM-based technique, allowing smaller distances to be probed. Another difference among the two sets of data was the slope of the plateaus; they were steeper in the case of the STM-BJ. However, this can also be a consequence of a difference in calibration or due to the different speeds used in the two techniques; at this point, no clear conclusion can be drawn from this observation. Interestingly, the STM-BJ experiments clearly showed a difference in the case of cysteine when changing the pH of the solution, and different conductance values and classes appeared (see purple versus brown sloped line segments in Figure 5). Lastly, when comparing the methionine conductance values with the one reported in Reference [11], all three setups shared the conductance value near 10^‒3^ G_0_.

### 3.2. Theoretical Calculations

In order to shed light on the experimental results and the presence of various conductance plateaus in particular, we performed theoretical calculations based on a combination of DFT and NEGF techniques, as described in Section 2.

**Figure 6:**
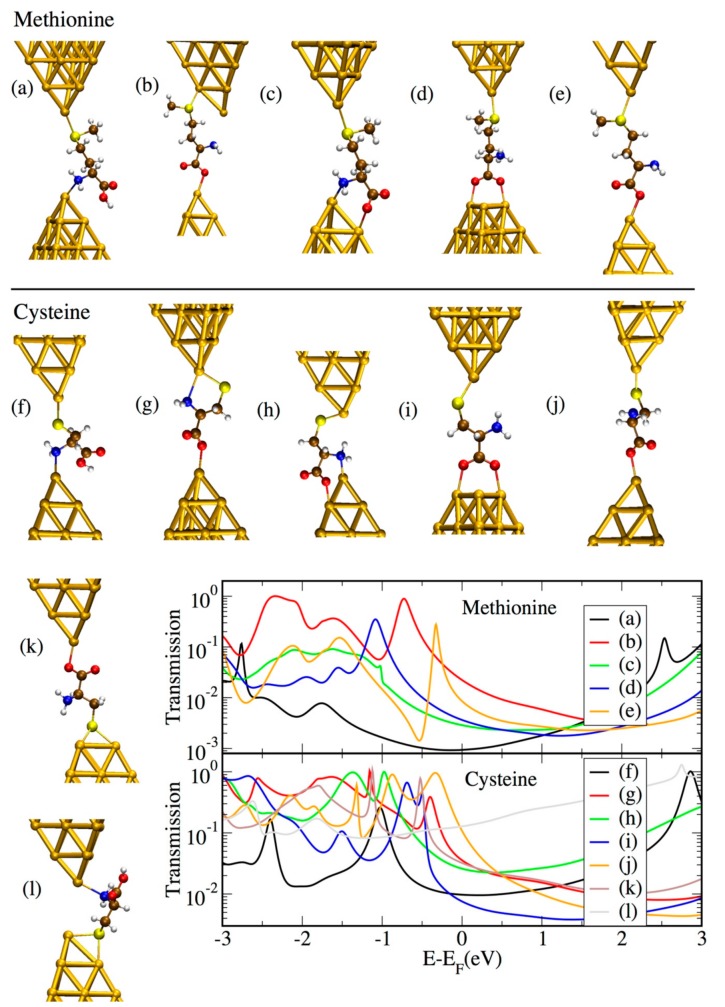
Analyzed geometries for junctions incorporating methionine (**a**–**e**) and cysteine (**f**–**l**) and corresponding transmission curves.

The majority of the amino acids studied in this work possess more than two groups available for binding to the gold electrodes within their structure. This is likely to give rise to various conductance plateaus during stretching of the junction, consistent with the experimental observations. As an example, we showed here several possible binding configurations for methionine (Figure 6a–e) and cysteine (Figure 6f–l), together with the corresponding transmission curves. These structures show possible binding scenarios which originate from the presence of three different anchor groups (thiol, carboxyl, and amino) and their possible coordination. Note, that the type of binding formed for cysteine in Figure 6k–l was not considered for methionine since the –SMe group is known to favor binding to undercoordinated gold atoms [30,31]. For the cases in which the molecule was bound to gold via the –COOH group (as in Figure 6b–e,g–k), the hydrogen was removed according to previous literature [32,33,34,35,36,37]. However, since it has also been claimed that the occurrence of the H desorption depends upon the pH condition [38], we further considered the case in which the H ion was preserved: in this case, a shift to lower energy was observed (see Figure S9 of the Supplementary Materials). It is also worth noting that these geometries represent a limited set from the many possible ones. Others are indeed plausible corresponding to different elongation stages of the junction and which are not considered here for computational reasons.

All geometries correspond to conductance values which span a range of less than two orders of magnitude, making them probably hard to distinguish in the experiments. In the case of cysteine, for geometry “l”, the transmission is much higher than for several other geometries over a broad energy range around the Fermi energy. This is caused by the short Au–Au distance which, in turn, causes the transmission curve to contain a considerable contribution from direct Au–Au tunneling. It is also possible to observe that geometries in which binding takes place at the nitrogen of the NH_2_ group tend to give rise to a shift in the molecular orbitals to lower energies for both cysteine and methionine (Figure 6a,c,h,f,l). Geometry “g” represents an exception to this trend, probably because of the additional binding through S on the same side of the junction as for NH_2_. The observation of a lower energy alignment for NH_2_ compared to S binding is consistent with previous literature [39]. Incorporating cysteine in molecular junctions has been claimed to give rise to negative differential resistance (NDR) [17] because of its strongly asymmetric structure. However, this has not been considered in the present study, where the experiments have been carried out at low bias, ruling out this effect. For geometry “e” of methionine, an antiresonance–resonance pair can be observed close to the Fermi level, which stems from the interference among two orbitals (which coincide with the HOMO-1 and HOMO-2 of the Au1–methionine–Au1 system shown in Figure S12). 

It is important to bear in mind that the computed conductance might be overestimated due to the well-known HOMO–LUMO gap underestimation in DFT. In fact, the transmission resonances corresponding to the occupied states close to the Fermi level might actually lie at lower energies than in the DFT results. Moreover, even lower conductance values could be obtained by the possible formation of either dimers [40] or gold thiolate units [31,41] (see Figure S8). The role of the surrounding water molecules in the experiments should also be taken into account, as it could also induce a decrease in conductance (see Figure S10). Despite the uncertainties in the DFT, which prevent us from making a quantitative comparison between theoretical and experimental results; the high variation of transmission values in the energy region around the Fermi level indicates that the conductance of cysteine- and methionine-based junctions in the experiments is likely to be ambiguous.

**Figure 7:**
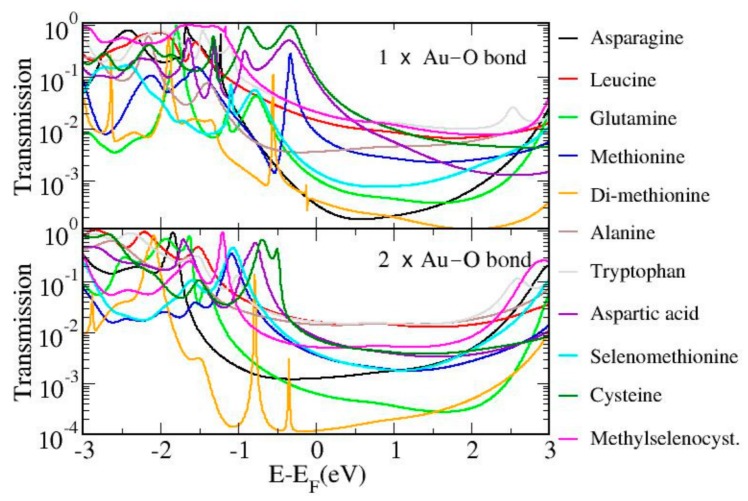
Transmission as a function of energy for all amino acids considered in the “1 × Au–O bond” and “2 × Au–O bond” structures.

We now turn to the comparison of the other amino acids studied in the experiments. As mentioned above, all contain more than two anchor groups in their backbone. Consequently, and similarly to methionine and cysteine, they are expected to provide a wide range of possible conductance values. However, since performing the same analysis for them all as for the two aforementioned cases is computational demanding, we instead focused on one specific case, namely, the one in which they all bind to gold via the common COO– group on one side. The anchor group on the other side changes depending on the amino acid and it has generally been chosen as the group located at the opposite end of the backbone with respect to COO–, thus –S for cysteine and methionine, –O for aspartic acid, –Se for selenomethionine and methylselenocysteine, and –N for tryptophan, Di-methionine, alanine, asparagine, and glutamine. For most amino acids studied here, this choice corresponds to analyzing the fully elongated structure. This anchor-group selection, however, does not include moieties which are known to bind to gold via physisorption (present in leucine and tryptophan, for instance). For this comparison, we also avoided configurations in which the amino acid was bound to gold through more than one group as in structure “g” of cysteine in Figure 6. Furthermore, we consider two types of binding between the COO– and Au: one in which it binds via only one O–Au bond (as in structures “e” and “j” of Figure 6) and one in which it binds through two O–Au bonds (as in structures “d” and “i” of the same figure). These two geometries will henceforth be called 1 × O–Au bond and 2 × O–Au bond, respectively. In Figure 7, we show the transmission curves for both geometries.

We found that for each configuration, transport takes place through either the HOMO or the HOMO-1. The two sets did not show exactly the same trends in conductance through all amino acids considered (see below for further details). This is probably because the different orientations of the COO– group with respect to the junction direction in the two geometries induces different geometrical rearrangements. Such structural modifications are favored by the rather flexible structure of these systems. Considering a much larger number of geometries for each amino acid throughout the whole elongation process would be preferential, in order to take into account different levels of strain in the junction as well as binding through the other anchor groups contained in the backbone (including those which bind to gold via physisorption, which has been proposed as a possible scenario in previous work [8]). This computationally demanding extension of the analysis would be desirable in future studies and could for instance shed more light on the slanted plateau shapes observed in experiment.

We now turn to the comparison of the transmission curves of the two sets in detail. For most amino acids, the resonance corresponding to the HOMO lies at lower energy in the 2 × O–Au bond geometry than in the 1 × O–Au bond geometry. The amino acids connected to gold at one end via –S (cysteine, methionine), Se (selenomethionine, methylselenomethionine) and –O (aspartic acid) appear to yield resonances closer to the Fermi level as compared to the amino acids connected to gold via –N (tryptophan, alanine, asparagine, glutamine, and leucine). An exception to this trend is di-methionine, which is connected via –N and the HOMO of which lines up not far from the Fermi level; however, the different behavior is not surprising given the quite different structure as compared to the other amino acids. In fact, this molecule shows very narrow peaks and generally lower conductance than the others in both geometries. It is worth mentioning that, for this molecule, the two geometries considered in Figure 7 did not correspond to the junction of maximum length. The latter was actually obtained by binding through the S atoms at both ends which yielded much lower conductance (see Figure S11 in the Supplementary Materials).

The energy alignment observed in the junction for the amino acids considered did not follow the same trend as for the energetic position of the gas-phase HOMO (the energy values for the gas-phase frontier orbitals for all systems are reported in Supplementary Materials Section 10). This suggests that the alignment in the junction is strongly influenced by the anchoring group available for binding in each amino acid. Moreover, the trend observed in the energy alignment did not seem to be correlated with the conductance values. For instance, leucine showed a higher conductance than many other amino acids despite its orbital low-energy alignment: this is mainly caused by the higher broadening on the HOMO-related peak as compared with the other systems. However, it is also important to note that the broadening of each resonance was found to depend on the strain on the junction, which was not the same for all geometries considered in Figure 7.

Quantitative conclusions about the comparison between theory and the experiments cannot be drawn as a result of the uncertainties in the DFT energy alignment of the orbitals. Indeed, the robustness of these results should be tested in future studies by use of different exchange-correlation functionals, which were shown to improve the description of electronic properties of several systems [42,43,44,45]. The number of geometries considered throughout the whole series of amino acids, moreover, is limited. Thus, it is difficult to establish a correspondence between the many possible binding structures which were discussed above and those analyzed in the experiments, where several kinds of configurations probably take place throughout the junction elongation. For instance, in Reference [11], it was suggested that aspartic acid is most likely to bind to gold via the N atom of the amino group, which has not been considered here. Gauche defects have also been shown to affect the conductance [46] and the possible role of water discussed in Figure S10 of the Supplementary Materials should also be considered. Nevertheless, the above results lead us to conclude that the conductance of each amino acid is given by a delicate interplay between the energy alignment induced by the anchoring groups used in a specific structure, its length, the degree of hybridization of the relevant orbital and the possible conformational changes (gauche defects and different degree of elongation or compression). These factors are particularly important in the case of these molecules because of their flexibility and the presence of more than just two anchoring groups in their structure.

In summary, the theoretical conductance values obtained for the amino acids considered are higher than the experimental values due to the well-known uncertainties in DFT. This hinders a quantitative comparison. What can be learned instead from the theoretical results can hence be summarized in the following points: (i) amino acids can bind to gold through a large variety of configurations, which leads, in the case of cysteine and methionine, to conductance values which span a range larger than one order of magnitude: this agrees with the experiments (Figure 4) and confirms that the variety of conductance groups measured originates from the broad variety of possible binding structures; (ii) the energetic position of the molecular orbital relevant for the electron transport depends on the group used for anchoring in each amino acid; the electrical conductance is then a result of this effect, combined with the length of the amino acid and finally modulated by the metal–molecule coupling.

## 4. Conclusions

We have performed STM-BJ and MCBJ measurements on molecular junctions based on ten amino acids and one di-methionine dimer, finding several conductance groups for each. The DFT-based calculations helped in identifying the origin of the broad variety of these groups, which is the possible formation of various kinds of binding configurations between the molecules and the metal electrodes and the possible role of other factors such as conformational changes and pH changes. The difficulty in establishing a clear correspondence between the measured conductance values and the exact type of molecular junctions suggests further research. More systematic studies in which only one parameter is changed would be helpful, as well as the incorporation of strategies which favor particular binding configurations, e.g., such as those used in recognition tunneling experiments [12,47]. In addition, further theoretical calculations should focus on the conductance evolution while stretching, as well as taking thermal fluctuations into account.

## Supplementary Materials

The following are available online at https://www.mdpi.com/2218-273X/9/10/580/s1, Figure S1 (Section 1): Exemplary traces for methionine and cysteine, measured with the MCBJ and STM-BJ setup; Figure S2–S6 (Section 2): Clustering method and the 1D and 2D of all classes for methionine and cysteine; Figure S7 (Section 3): Stability of the clustering method; Section 4: Preparation of the data. Table S1–S2 (Section 5): Overview over the positions and yields of the 5 classes; Section 6: Amino acid suppliers; Figure S8 (Section 7): Cysteine dimers and gold thiolates; Figure S9 (Section 8): The role of the H ion of the COOH group; Table S3 (Section 10): Gas-phase energy levels. Figure S10 (Section 9): The effect of water; Figure S11 (Section 11): Di-methionine: additional geometries; Figure S12 (Section 12): Spatial distribution of the frontier orbitals.
