# Supplementary material for: Can One Define the Conductance of Amino Acids?
Source: Biomolecules. 2019 Oct 7;9(10):580. doi: 10.3390/biom9100580 (PMC6843363; doi:10.3390/biom9100580)
Supplement: Supplementary file 1 [file biomolecules-09-00580-s001.pdf]

Supplementary Information for

Can one define the conductance of amino acids?

Linda A. Zotti,[1,4]* Beatrice Bednarz,[2] Juan Hurtado-Gallego,[3] Damien Cabosart,[2] Gabino-Rubio Bollinger,[3,4] Nicolas Agrait[3,4,5] and Herre S.J. van der Zant[2,*]

[1] Departamento de Física Teorica de la Materia Condensada, Universidad Autońoma de Madrid, Ciudad Universitaria de Cantoblanco, E-28049 Madrid, Spain

[2] Kavli Institute of Nanoscience, Delft University of Technology, Lorentzweg 1, 2628 CJ Delft, The Netherlands

[3] Departamento de Física de la Materia Condensada, Universidad Autońoma de Madrid, Ciudad Universitaria de Cantoblanco, E-28049 Madrid, Spain

[4] Condensed Matter Physics Center (IFIMAC), Universidad Autońoma de Madrid, Ciudad Universitaria de Cantoblanco, E-28049 Madrid, Spain

[5] Instituto Madrileño de Estudios Avanzados en Nanociencia (IMDEA Nanociencia), Campus Universitario de Cantoblanco, E-28049 Madrid, Spain

* Correspondence: linda.zotti@uam.es, H.S.J.vanderZant@tudelft.nl

Table of Contents

Experiment:

1. Exemplary breaking traces for methionine and cysteine, measured with the MCBJ and STM-BJ setup

2. Clustering method and the 1D and 2D histograms of all classes for methionine and cysteine

3. Stability of the clustering method

4. Preparation of the data

5. Overview over the positions and yields of the 5 classes

6. Amino acid suppliers

Theory:

7. Cysteine dimers and gold thiolates

8. The role of the H ion of the COOH group

9. The effect of water

10. Gas-phase energy levels

11. Di-methionine: additional geometries

12. Spatial distribution of the frontier orbitals

1. Exemplary traces for methionine and cysteine, measured with the MCBJ and STM-BJ setup

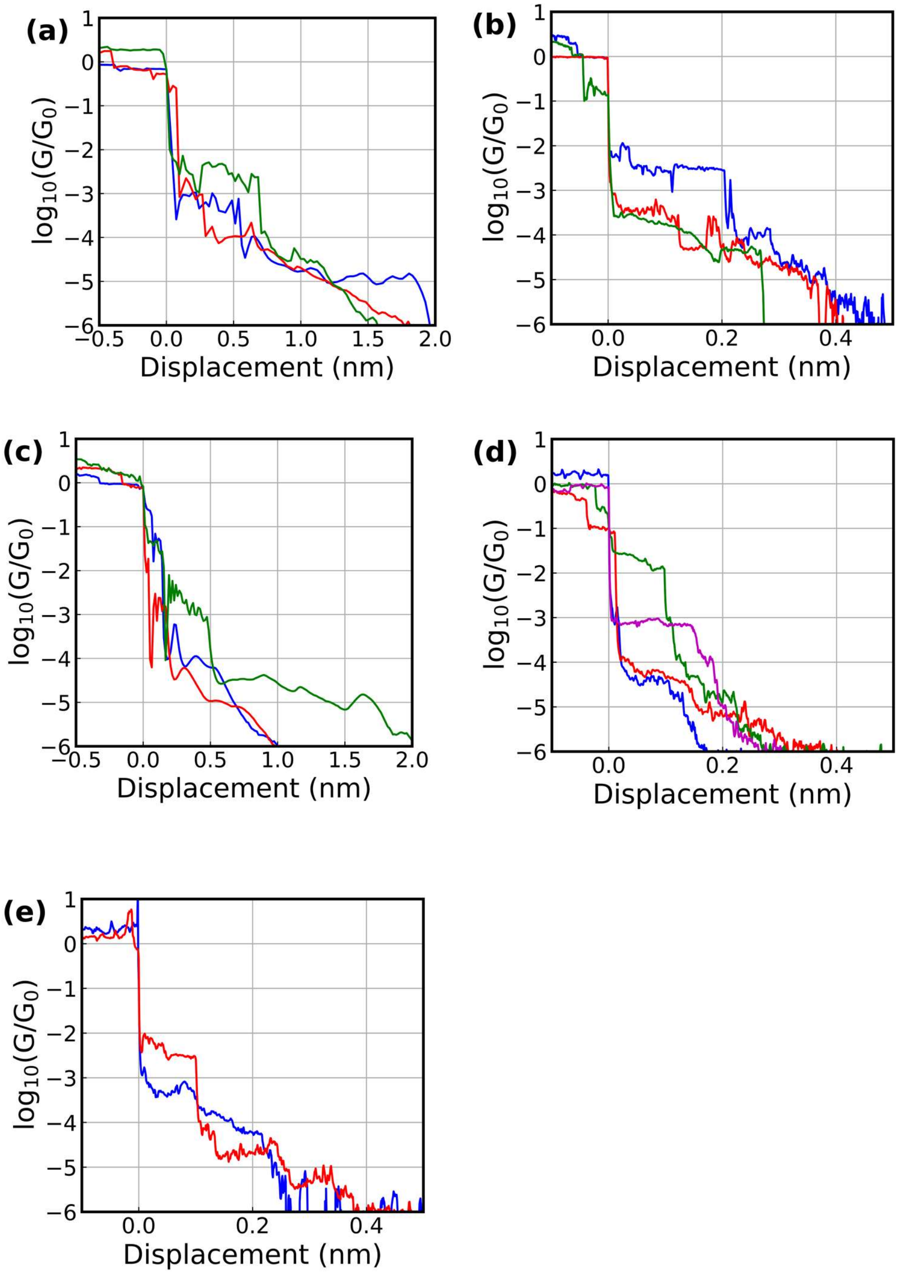

Figure S1: Individual traces from different classes with plateaus for (a) methionine measured with the MCBJ setup, (b) methionine measured with the STM-BJ setup, (c) cysteine measured with the MCBJ setup, (d) cysteine measured at pH 7 with the STM-BJ setup and (e) cysteine measured at pH 10 with the STM-BJ setup.

2. Clustering method and the 1D and 2D histograms of all classes for methionine and cysteine

The goal of the clustering analysis is to sort the breaking traces into groups which share the same plateaus. The K-means clustering algorithm is an unsupervised and reference-free learning tool which is able to analyse a high dimensional space.[1] To compare the breaking traces, we created a binned image of each and compared the height profiles. Each of the bins is referred to as a feature. Additionally, 1D histograms were created by adding up all bins at a fixed conductance. This information was added as extra features to the features of the corresponding binned image. As inputs, K-means needs the feature vector for every trace and the number of clusters into which it should group the traces. The optimal groups found by K-means are referred to as classes.

Every class can then be analysed individually. First, the 1D and 2D histogram of the traces inside the class were generated. From the 1D histogram it was determined whether the class has plateaus. If yes, their position G_i and standard deviation $\sigma(G_i)$ were obtained by fitting the peak in the 1D histogram with a Gaussian. At these positions, all traces within the class were analyzed to find the average length and slope of the plateau.

To find the plateau length, the data points inside the conductance regime $[G_i - \sigma(G_i), G_i + \sigma(G_i)]$ were detected in every trace. The plateau length was taken to be the distance between the first and last one of these points. The length was averaged over all traces and the standard deviation was computed. Including some points outside the plateau, does not change the obtained plateau length considerably, because the conductance drops fast at the plateau ends.

To obtain its slope, the plateau was fitted with a linear dependence for every trace. The plateau slopes are not normally distributed and have outliers far off the average slope. Therefore, the median of the slopes was computed. For the error, the two slopes were determined for which 20% of the values are below and 80% were above, respectively. The difference to the average slope was computed and the two differences averaged. Additionally, the tunnelling slope was fitted for every curve between conductance values of 10^{-5} and 10^{-6} G_0 for comparison.

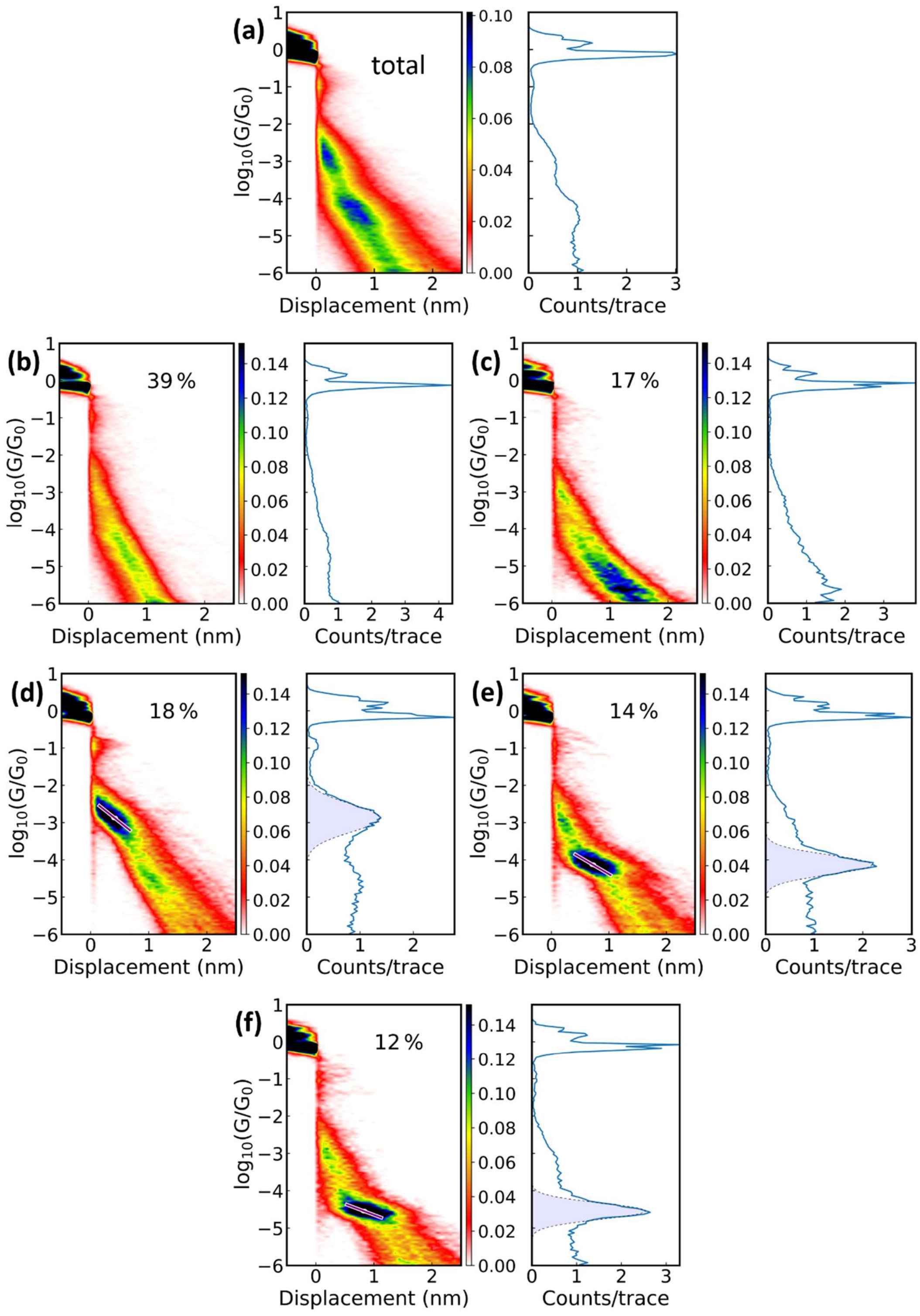

Figure S2: 1D and 2D histograms of the raw data of 6,000 breaking traces of methionine, measured at 0.1 V bias voltage with the MCBJ setup. (a) The complete dataset and (b-f) the results of the clustering analysis into 5 classes are shown. The lines in the 2D histograms of figures (d-f) are the plateau fits with their centre at the peak position of the 1D histogram. The fits are shown in figure 4 of the main text.

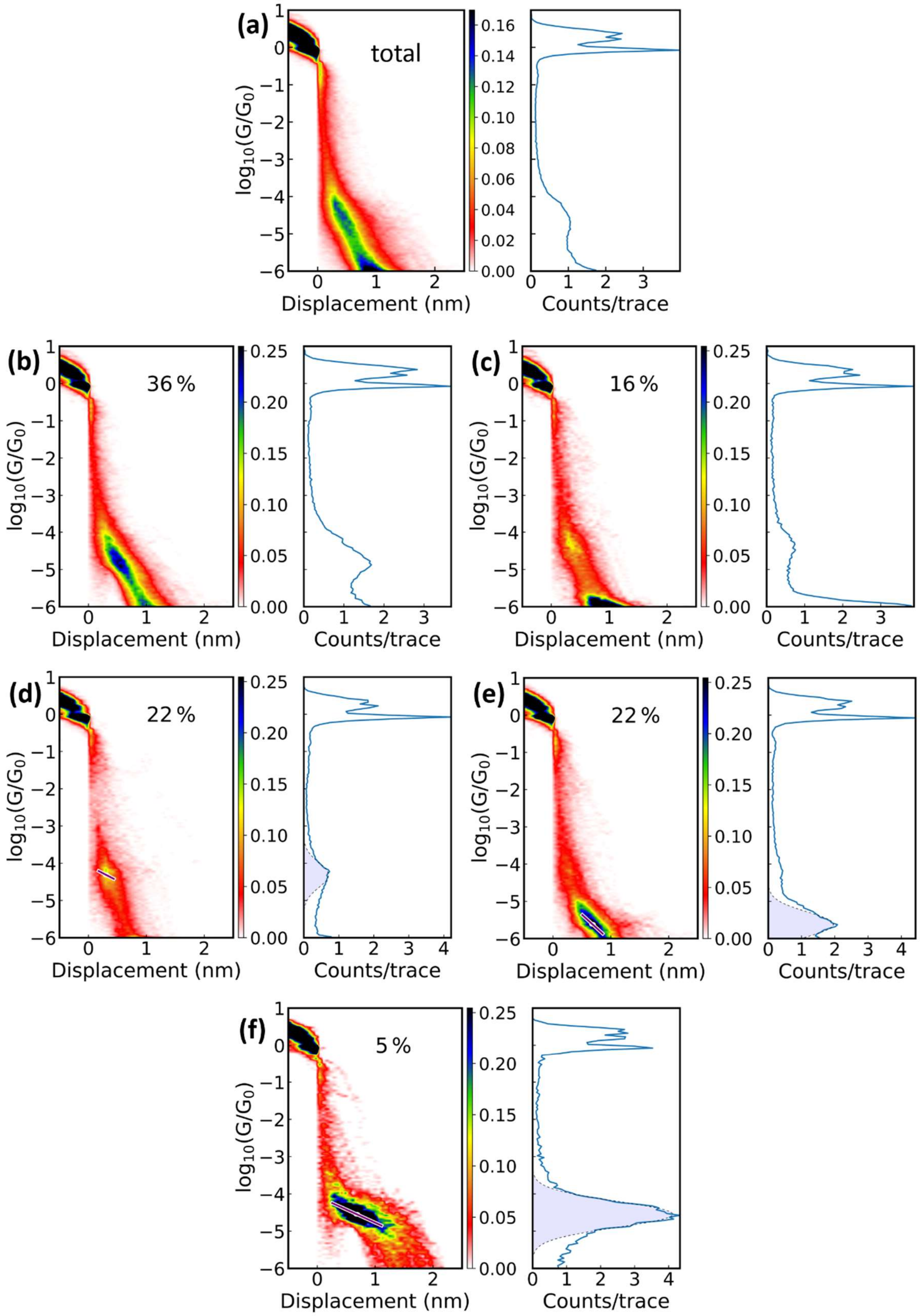

Figure S3: 1D and 2D histograms of the raw data of 4,000 breaking traces of cysteine, measured at 0.1 V bias voltage with the MCBJ setup. (a) The complete dataset and (b) the results of the clustering analysis into 5 classes are shown. The lines in the 2D histograms of figures (d-f) are the plateau fits with their centre at the peak position of the 1D histogram. However, the plateau in figure (f) is, with (0.9 ± 0.3) nm, longer than the molecule. Therefore, it was discarded and is not shown in figure 4.

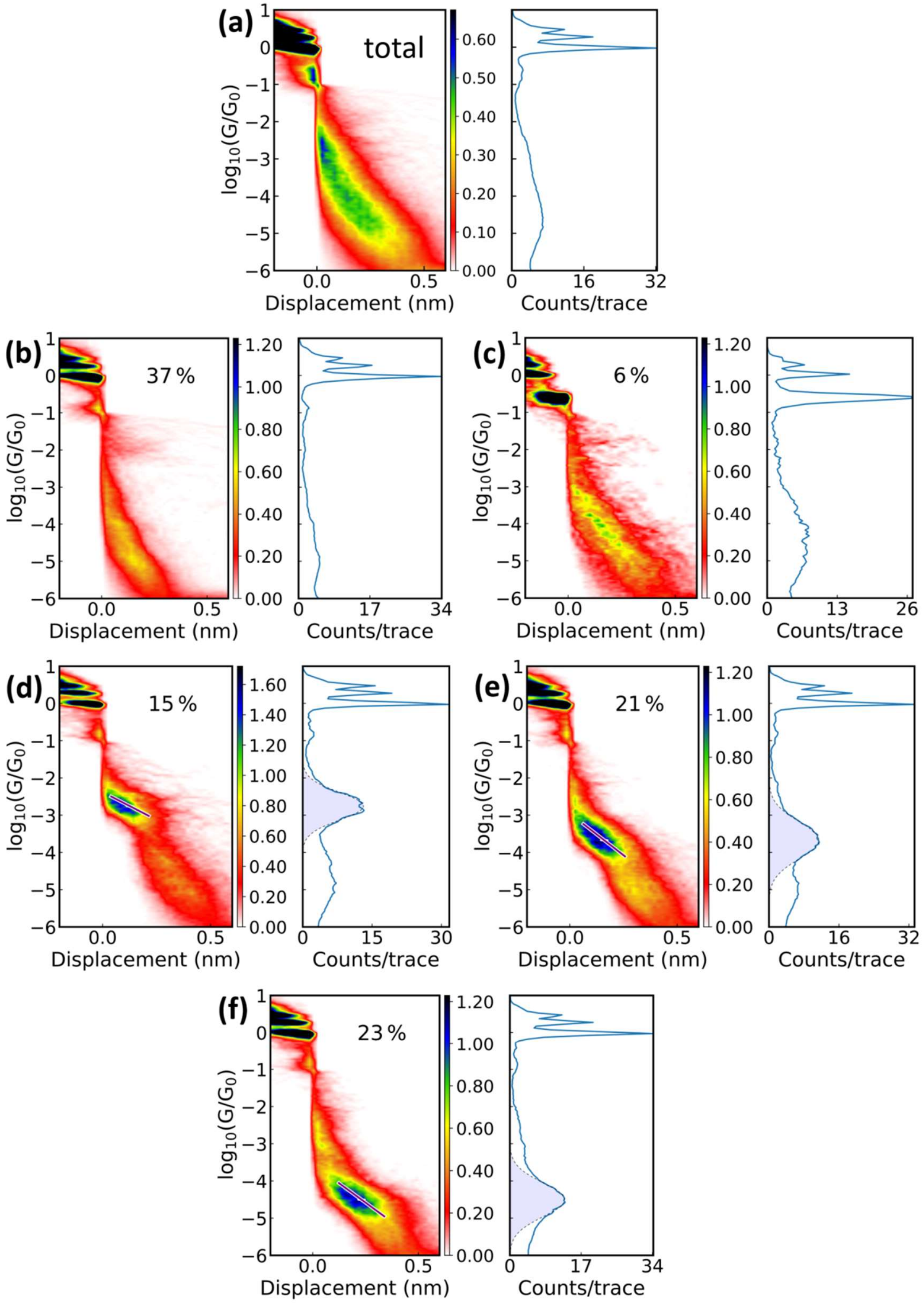

Figure S4: 1D and 2D histograms of the raw data of 5,745 breaking traces of methionine measured with the STM-BJ. (a) The complete dataset and (b) the results of the clustering analysis into 5 classes are shown. The lines in the 2D histograms of figures (d-f) are the plateau fits with their centre at the peak position of the 1D histogram.

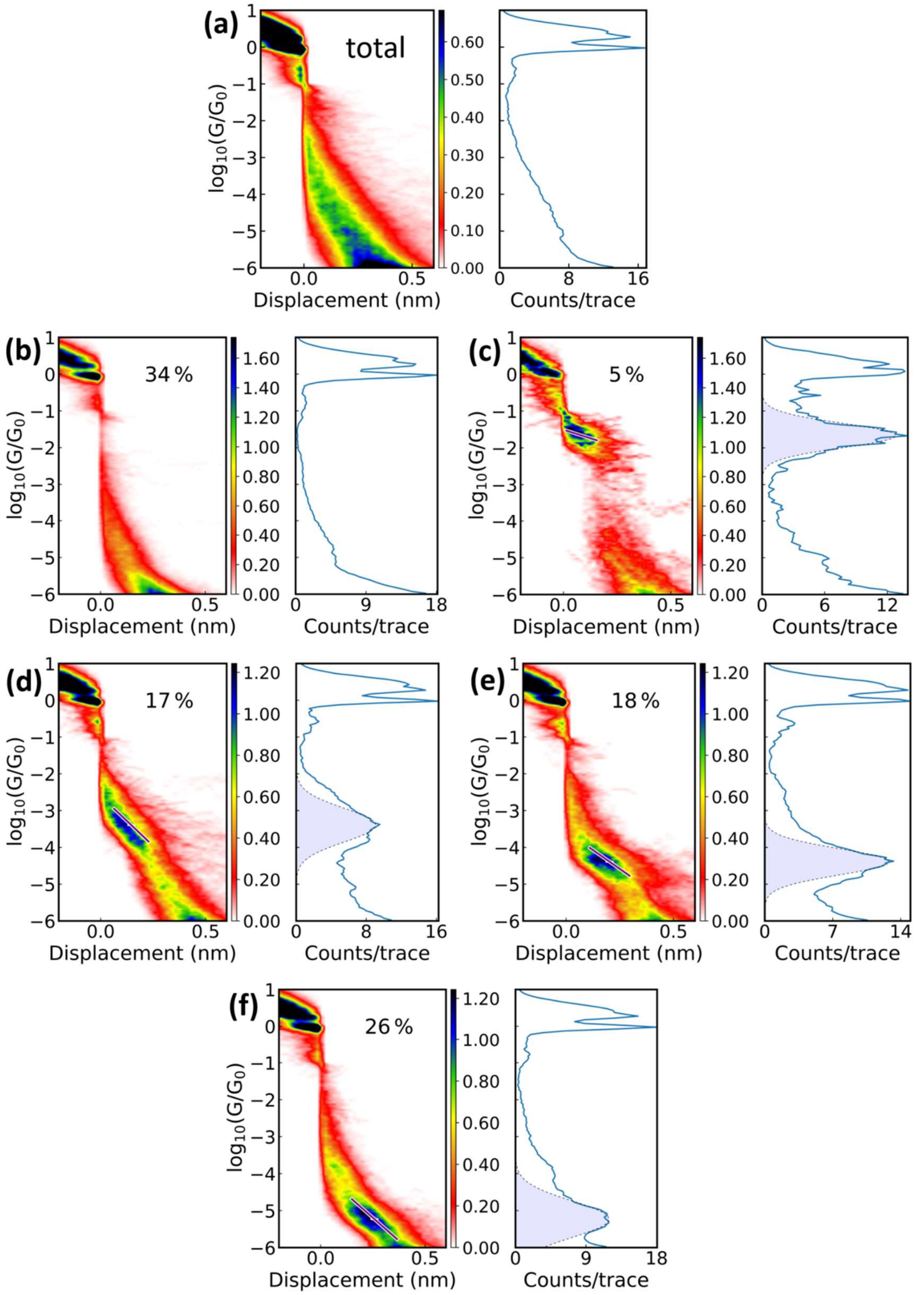

Figure S5: 1D and 2D histograms of the raw data of 3,584 breaking traces of cysteine which was dissolved in a solution of pH 7 and measured with the STM-BJ. (a) The complete dataset and (b) the results of the clustering analysis into 5 classes are shown. The lines in the 2D histograms of figures (d-f) are the plateau fits with their centre at the peak position of the 1D histogram.

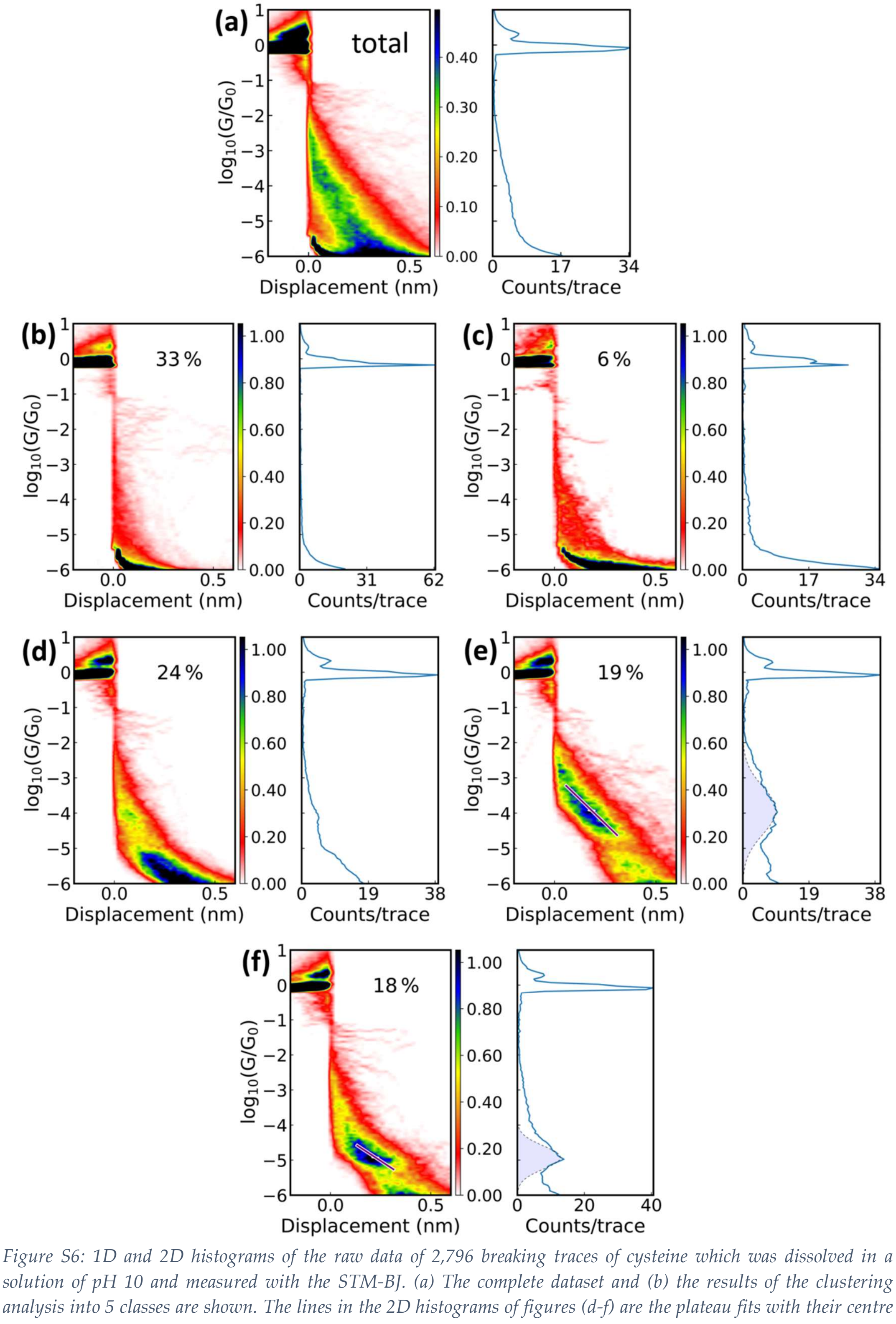

Figure S6: 1D and 2D histograms of the raw data of 2,796 breaking traces of cysteine which was dissolved in a solution of pH 10 and measured with the STM-BJ. (a) The complete dataset and (b) the results of the clustering analysis into 5 classes are shown. The lines in the 2D histograms of figures (d-f) are the plateau fits with their centre at the peak position of the 1D histogram.

3. Stability of the clustering method

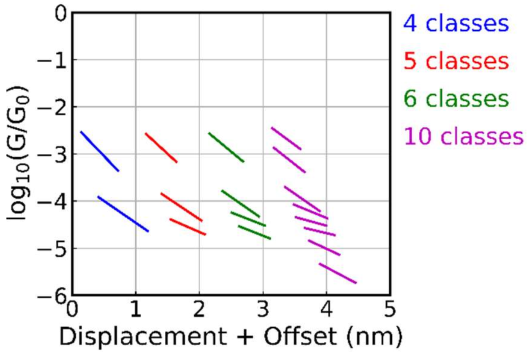

Figure S7: Comparison of the average plateau positions from clustering 6,000 methionine traces into 4, 5, 6 and 10 classes, respectively. Plateaus were only considered, if they were shorter than the molecule and less steep than the tunnelling slope.

A very important input parameter of the clustering method is the number of groups that the clustering has to yield. Figure S7 shows how the number of detected plateaus changes depending on the chosen number of clusters. If the data is divided into more classes, more plateaus at different positions will be found. Partly, new plateaus appear, like the ones at a conductance below 10^{-5} G_0 for 10 classes. Furthermore, existing clusters tend to split up into two. It can be true, that they actually correspond to two plateaus (corresponding to distinctively different junction configurations) that are lying close together, but it can also happen, that one plateau gets split up into two, while representing a very similar junction configuration. Conclusions can therefore only be drawn about whether plateaus exist in a certain conductance regime, not about how many plateaus there are exactly. Five classes were chosen as an intermediate number to visualize the conductance behaviour across the different molecules.

4. Preparation of the data

Before analysing the data, all traces were aligned such that the breaking starts at a displacement value of zero. Traces in which the conductance didn't drop to the noise level, meaning that the junction didn't break completely, were removed. Then, the noise level was subtracted. The displacement-axis was calibrated using the tunnelling slopes of the pure gold. Since the measurements are performed in air, work function values around (1.0 ± 0.2) eV were used in the fitting.[2,3] This corresponds to a tunnelling slope of approximately -4.4 nm^{-1} on the decadic logarithmic scale to which we calibrated all measurements.

5. Overview over the positions and yields of the 5 classes

Table S1: Yields and plateau positions of a clustering analysis with K-means into 5 classes for the molecules measured with the MCBJ. Plateaus are only considered, if they are shorter than the amino acid and less steep than the tunneling slope.

		**Class 1**	**Class 2**	**Class 3**	**Class 4**	**Class 5**
**Alanine**	Yield	28 %	21 %	19 %	15 %	17 %
	G_{peak} / G_0	/	/	/	$(6.3 \pm 1.0) \cdot 10^{-5}$	$(2.9 \pm 0.8) \cdot 10^{-5}$
**Leucine**	Yield	36 %	32 %	25 %	3 %	4 %
	G_{peak} / G_0	/	$(4.6 \pm 1.4) \cdot 10^{-5}$	/	/	/
**Aspartic acid**	Yield	33 %	23 %	15 %	15 %	14 %
	G_{peak} / G_0	/	/	/	$(7.7 \pm 1.5) \cdot 10^{-5}$	$(4.1 \pm 0.8) \cdot 10^{-5}$
**Glutamine**	Yield	30 %	21 %	15 %	12 %	21 %
	G_{peak} / G_0	/	$(6.6 \pm 1.5) \cdot 10^{-5}$	$(4.3 \pm 0.7) \cdot 10^{-5}$	$(3.2 \pm 0.5) \cdot 10^{-5}$	/
**Cysteine**	Yield	36 %	16 %	22 %	22 %	5 %
	G_{peak} / G_0	/	/	$(5 \pm 2) \cdot 10^{-5}$	$(2.4 \pm 0.8) \cdot 10^{-6}$	/
**Methionine**	Yield	39 %	17 %	18 %	14 %	12 %
	G_{peak} / G_0	/	/	$(1.3 \pm 0.5) \cdot 10^{-3}$	$(7 \pm 2) \cdot 10^{-5}$	$(2.8 \pm 0.6) \cdot 10^{-5}$
**Di-Methionine**	Yield	50 %	11 %	13 %	13 %	13 %
	G_{peak} / G_0	/	$(10 \pm 2) \cdot 10^{-6}$	$(6.3 \pm 0.9) \cdot 10^{-6}$	$(4.0 \pm 0.6) \cdot 10^{-6}$	$(2.5 \pm 0.4) \cdot 10^{-6}$
**Methyl-seleno-cysteine**	Yield	32 %	17 %	20 %	16 %	15 %
	G_{peak}/ G_0	/	$(24 \pm 5) \cdot 10^{-2}$	$(9 \pm 3) \cdot 10^{-2}$	$(28 \pm 6) \cdot 10^{-7}$	$(11 \pm 3) \cdot 10^{-7}$
**Seleno-methionine**	Yield	43 %	13 %	14 %	14 %	16 %
	G_{peak} / G_0	/	$(28 \pm 4) \cdot 10^{-2}$	$(17 \pm 3) \cdot 10^{-2}$	$(9 \pm 2) \cdot 10^{-2}$	$(3.3 \pm 1.0) \cdot 10^{-2}$
**Tryptophan**	Yield	22 %	30 %	17 %	17 %	14 %
	G_{peak} / G_0	/	/	$(9 \pm 2) \cdot 10^{-6}$	$(4.8 \pm 0.9) \cdot 10^{-6}$	$(2.8 \pm 0.5) \cdot 10^{-6}$

Table S2: Yields and plateau positions of a clustering analysis with K-means into 5 classes for the molecules measured with the STM-BJ. Plateaus are only considered, if they are shorter than the amino acid.

		Class 1	Class 2	Class 3	Class 4	Class 5
**Cysteine, pH 7**	Yield	34 %	5 %	17 %	18 %	26 %
	G_{peak} / G_0	/	$(2.2 \pm 0.7) \cdot 10^{-2}$	$(4 \pm 2) \cdot 10^{-4}$	$(4.1 \pm 1.5) \cdot 10^{-5}$	$(7 \pm 3) \cdot 10^{-6}$
**Cysteine, pH 10**	Yield	33 %	6 %	24 %	19 %	18 %
	G_{peak} / G_0	/	/	/	$(1.1 \pm 0.8) \cdot 10^{-4}$	$(1.2 \pm 0.4) \cdot 10^{-5}$
**Methionine**	Yield	37 %	6 %	15 %	21 %	23 %
	G_{peak} / G_0	/	/	$(1.8 \pm 0.7) \cdot 10^{-3}$	$(2.1 \pm 1.0) \cdot 10^{-4}$	$(3.2 \pm 1.5) \cdot 10^{-5}$

6. Amino acid suppliers

Amino acids have been obtained from the following suppliers:
Alanine: Alfa Aesor
Asparagine: Sigma Aldrich
Aspartic acid: Fluka
Cystine: Fluka
Dimethionine: Alfa Chemistry
Glutamine: Sigma Aldrich
Leucine: Sigma Aldrich
Methionine: Sigma Aldrich
Methylselenocysteine: Sigma Aldrich
Tryptophan: Fluka
Selenomethionine: Thermo Fisher

7. Cysteine dimers and gold thiolates

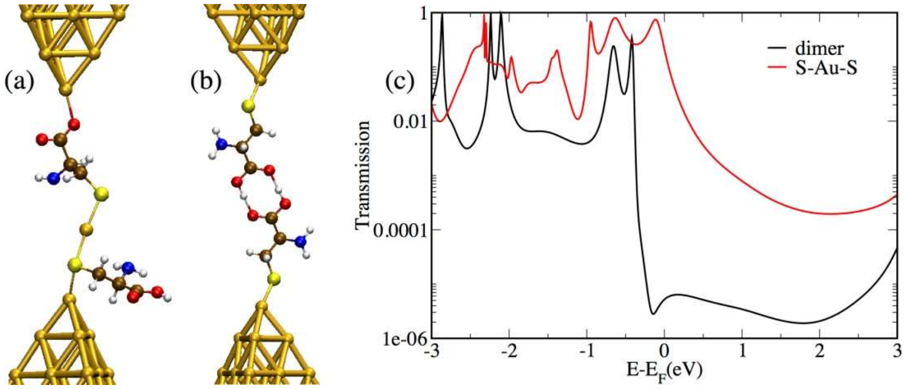

Figure S8: Au-cysteine-Au structures in which a gold thiolate unit has been formed due to the trapping of a gold atom between two neighbouring molecules (left) and in which a dimer has been formed (center); the right-hand panel shows the corresponding transmission curves.

Besides the Au-cysteine-Au junctions considered in Figure 6 of the main text, we considered two more cases. Figure S8 displays the trapping of a gold atom between two neighboring molecules; this was suggested to be a plausible effect in break-junction measurements in the presence of thiolated molecules[4,5] and was claimed to give rise to conductance values much lower than for a standard Au-molecule-Au juntion. In the case of the cysteine-based junction considered here, the HOMO-related peak unfortunately lies quite close to the Fermi energy, corresponding actually to quite a high transmission at the Fermi level. However, given the already-mentioned inaccuracy of the HOMO-LUMO gap, this peak is most likely to lie at lower energy. Consequently, in view of the much lower transmission produced by this geometry in the energy region immediately above the Fermi level, one can foresee that the real gap would actually result in a much lower conductance.
We now turn to discuss Figure S8b, where we have instead considered the formation of a dimer, the possible formation of which was suggested in Ref.[6]. This configuration clearly gives rise to a very low conductance (much lower than any of the other geometries considered for cysteine). The transmission curve also shows an interference feature close to the Fermi level; this in turn stems from the two highest occupied orbitals of the dimer (which are degenerate).

8. The role of the H ion of the COOH group

In the next figure (Fig. S9), we show a comparison between the transmission curves obtained for cysteine and methionine with and without the H atom of the COOH group. These two cases will be henceforth referred to as COOH and COO-. To this aim, we chose geometry "g" and "e" of Figure 6 of the main text for cysteine and methionine, respectively. All the structures were optimized as described in the "Method" section of the manuscript. For both molecules, in the transmission curve of the COOH geometries the LUMO appears closer to the Fermi energy as compared to the COO- case. For methionine, we also note that the interference feature disappears as a result of a change in the metal-molecule coupling.
It is also worth mentioning that attempts to perform such a comparison for cysteine in geometry "j" (which is more similar to the structure "e" of methionine than "g" is) failed. This is because the presence of the additional H atom made the cysteine-gold bond at the O terminal weaker, causing the COOH-Au bond to break. Only by reducing the Au-Au distance considerably was the optimization possible, which led to the final geometry being the same as structure "g", i.e., with the formation of a NH_2-Au bond.

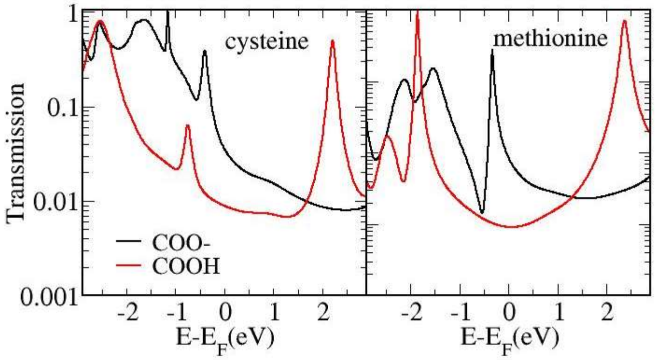

Figure S9: Transmission as a function of energy for cysteine in geometry "g" (left) and methionine in geometry "e"(right) of Figure 6 of the main with (red) and without (black) the H atom of the carboxyl group.

9. The effect of water

In order to understand how the presence of the water molecules surrounding the junctions would affect the electrical conductance, we analyzed a simple case in which a water molecule is placed in proximity of the NH_2 group of a cysteine- and a methionine-based junction. The

corresponding geometries are shown in the figure below (Fig. S10), together with the transmission curves for the structures with and without the presence of the water molecule. In both cases, the water causes the molecular resonance which is closer to the Fermi level to narrow down and to shift to lower energies, causing in turn a decrease in conductance. In order to reproduce the real experimental scenario, one should actually include a much higher number of water molecules.

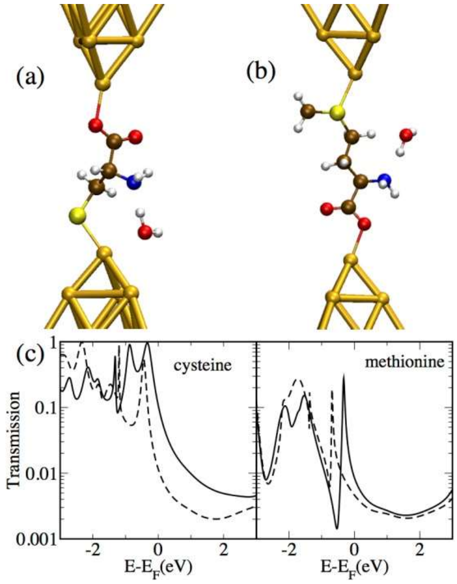

Figure S10: Junctions incorporating cysteine (a) and methionine (b) in the presence of a water molecule placed in proximity of the NH_2 group and transmission as a function of energy (c) with (dashed line) and without (solid line) the presence of the water molecule.

10. Gas-phase energy levels

In the following table we report the energy values (eV) for the Kohn-Sham HOMO and LUMO.

Table S3 Energy position (eV) for the Kohn-Sham HOMO and LUMO in the gas phase.

	**Alanine**	**Asparagine**	**Aspartic acid**	**Cysteine**	**Di-methionine**	**Glutamine**	**Leucine**	**Methionine**	**Methylselenocyst.**	**Selenomethionine**	**Tryptophane**
**HOMO**	-5.291	-5.249	-5.715	-5.440	-4.904	-5.416	-5.553	-5.001	-5.224	-4.826	-4.770
**LUMO**	-0.984	-1.350	-1.726	-1.176	-1.552	-1.277	-1.041	-1.123	-1.299	-1.123	-1.145

11. Di-methionine: additional geometries

In Fig. S11, we show the transmission curve for di-methionine for the "1 x O-Au bond" and "2 x O-Au bond" structures as well as a for a geometry in which it is connected to gold via a S atom on both sides. The latter case yields the lowest conductance as expected, given the longer length of this junction as compared to the other two geometries. The "1 x O-Au bond" case shows an interference feature, which stems from the presence of two competing conductance paths.

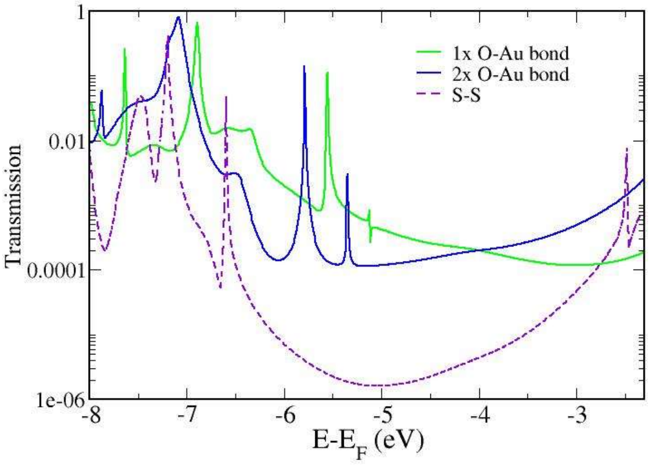

Figure S11: Transmission as a function of energy for di-methionine in the 1xO-Au bond, 2xO-Au bond and S-S geometries.

12. Spatial distribution of the frontier orbitals

Figure S12 shows the spatial distribution of the highest occupied orbitals for all amino acids studied in this work when connected to a gold atom on both sides as in the 1xO-Au geometries.

	HOMO-2	HOMO-1	HOMO
Alanine	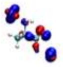	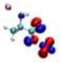	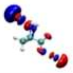
Asparagine	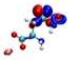	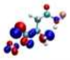	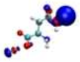
Aspartic acid	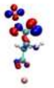	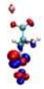	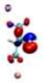
Cysteine	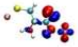	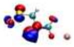	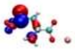
Di-methionine	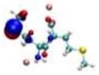	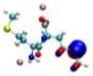	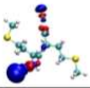
Glutamine	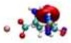	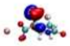	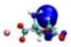
Leucine	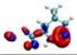	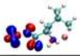	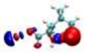
Methionine	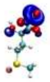	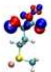	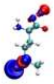
Methylselenocysteine	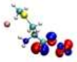	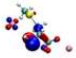	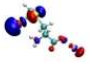
SelenoMethionine	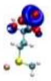	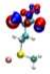	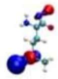
Tryptophane	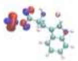	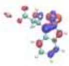	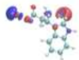

Figure S12: HOMO and LUMO for gas-phase molecules connected to one Au atom on each side. The Au atom is connected to the same anchor group as in the "1xO-Au bond" geometries and was here included in order to reproduce the same hybridization the molecular orbitals undergo when the amino acid is connected to gold clusters. The orbital corresponding to the main resonance close to the Fermi level is the HOMO-1 for all, except for cysteine and aspartic acid, for which is the HOMO.

References

1. Cabosart, D.; El Abbassi, M.; Stefani, D.; Frisenda, R.; Calame, M.; van der Zant, H. S.; Perrin, M. L. A Reference-Free Clustering Method for the Analysis of Molecular Break-Junction Measurements. *Appl. Phys. Lett.* **2019**, *114* (14), 143102.
2. Hong, Y. A.; Hahn, J. R.; Kang, H. Electron Transfer through Interfacial Water Layer Studied by Scanning Tunneling Microscopy. *J. Chem. Phys.* **1998**, *108* (11), 4367–4370. https://doi.org/10.1063/1.475847.
3. Hahn, J. R.; Hong, Y. A.; Kang, H. Electron Tunneling across an Interfacial Water Layer inside an STM Junction: Tunneling Distance, Barrier Height and Water Polarization Effect. *Appl. Phys. A* **1998**, *66* (1), S467–S472. https://doi.org/10.1007/s003390051184.
4. Leary, E.; Zotti, L. A.; Miguel, D.; Márquez, I. R.; Palomino-Ruiz, L.; Cuerva, J. M.; Rubio-Bollinger, G.; González, M. T.; Agrait, N. The Role of Oligomeric Gold–Thiolate Units in Single-Molecule Junctions of Thiol-Anchored Molecules. *J. Phys. Chem. C* **2018**, *122* (6), 3211–3218. https://doi.org/10.1021/acs.jpcc.7b11104.
5. Strange, M.; Lopez-Acevedo, O.; Häkkinen, H. Oligomeric Gold- Thiolate Units Define the Properties of the Molecular Junction between Gold and Benzene Dithiols. *J. Phys. Chem. Lett.* **2010**, *1* (10), 1528–1532.
6. Kühnle, A.; Linderoth, T. R.; Hammer, B.; Besenbacher, F. Chiral Recognition in Dimerization of Adsorbed Cysteine Observed by Scanning Tunnelling Microscopy. *Nature* **2002**, *415* (6874), 891.
